# Effect of dietary inclusion of hazelnut skin or hazelnut skin green extract on growth performance, carcass traits, and meat quality in heavy pigs

**DOI:** 10.3389/fvets.2026.1809078

**Published:** 2026-05-12

**Authors:** Katia D’Ambra, Roberta Trovato, Alice Cattivelli, Giovanna Minelli, Martino Musati, Claudio Forte, Silvia Tabasso, Davide Tagliazucchi, Domenico Pietro Lo Fiego

**Affiliations:** 1 Department of Life Sciences, University of Modena and Reggio Emilia, Reggio Emilia, Italy; 2 Interdepartmental Research Centre for Agri-Food Biological Resources Improvement and Valorisation (BIOGEST-SITEIA), University of Modena and Reggio Emilia, Reggio Emilia, Italy; 3 Department of Agriculture, Food and Environment, University of Catania, Catania, Italy; 4 Department of Veterinary Sciences, University of Turin, Grugliasco, Italy; 5 Department of Drug Science and Technology, University of Turin, Turin, Italy

**Keywords:** green phenolic extract, hazelnut skin, meat quality, oxidative stability, pig nutrition

## Abstract

**Introduction:**

Intensive livestock farming has sparked public debate, highlighting the need for sustainable livestock production. One effective strategy to improve both environmental sustainability and meat quality is the inclusion of agro-industrial byproducts in pig diets. This study aimed to investigate the effects of dietary inclusion of hazelnut skin (HS) or its green polyphenolic extract (HSE) on growth performance, carcass traits, and meat quality in heavy pigs.

**Methods:**

Seventy-two pigs were allotted to three dietary treatments (*n* = 24 each): a control group (C) fed a standard diet, and two experimental groups receiving the same diet, including either 0.6% HS replacing 0.6% of wheat bran (HSD) or adding 0.1% HSE (HSED). After growth performance assessment, 36 subjects, balanced by gender and treatment, were slaughtered for carcass and meat quality evaluations.

**Results:**

No differences were observed in growth performance or carcass yield, indicating all diets met nutritional needs. However, HSE supplementation increased thigh yield (*p* < 0.01). Overall, meat quality characteristics did not differ among treatments; however, HS inclusion showed positive trends in oxidative stability during cooking, n-3 polyunsaturated fatty acid content, and the n-6/n-3 ratio. HSE supplementation decreased cooking loss, giving it better water-holding capacity. The treated groups showed a reduction in cholesterol content in the meat of 7.56 and 12.10% in the HSD and HSED groups, respectively.

**Conclusion:**

Overall, the modest yet positive effects, combined with the absence of negative influences on growth performance, carcass traits, and meat quality, indicate that HS and HSE supplementation could offer commercial and environmental benefits in sustainable livestock systems.

## 1 Introduction

The quality of food, especially animal-derived products, is closely scrutinized by consumers, who are becoming more aware of the strong correlation between diet, health, and environmental sustainability (1). The environmental impact of intensive livestock farming has been a focal point of public debate for several years (2). However, despite rising concerns, a survey conducted among Norwegian consumers by Austgulen et al. (3) found that consumers may not yet be fully prepared to make dietary choices based on what is best for the climate or environment. Therefore, greater sustainability must primarily be achieved through more sustainable animal production systems. Feed production, in addition to being the main cost component of livestock farming in the EU, accounting for 55% of total costs in pigs in 2022 (4), also represents one of the major environmental burdens of the livestock sector. For pig production, feeds are responsible for the largest share of the environmental impact, contributing approximately 70% (5, 6). This significant impact is largely attributed to the global crop network used in feed production, which requires resources, energy, and long-distance transportation. A circular food system is seen as a solution for producing food within Earth’s limits by decoupling livestock feed from arable land to prevent competition for land resources. Currently, up to 40% of global arable land is used for livestock feed, with nearly half allocated to monogastric animals (7). Strategies such as using local ingredients and by-products from the food and bioenergy industries for feed are environmentally sustainable, provided they do not compromise growth performance (8). In 2015, the European Commission adopted an action plan for the circular economy aimed at reducing waste along the food supply chain and encouraging more sustainable practices, including the reuse of food waste as animal feed (9, 10). The incorporation of agro-industrial by-products into livestock feed not only supports the ecological transition of animal production systems but also provides functional benefits due to the presence of bioactive compounds. For instance, many of these by-products are rich in phenolic compounds, which exhibit strong antioxidant activity and are therefore valuable for improving feed preservation and enhancing meat quality (11). This antioxidant potential is particularly relevant for preventing the oxidation of polyunsaturated fatty acids in feed. Pork, for instance, is known for its favorable fatty acid profile, characterized by a high degree of unsaturation. However, this feature also increases its susceptibility to lipid oxidation, requiring the presence of bioactive compounds, such as polyphenol-derived metabolites with strong antioxidant capacity, in the tissues to enhance oxidative stability. Dietary supplementation represents the most direct and effective strategy to promote the accumulation of these compounds. Dietary phenolic compounds may be partially hydrolyzed and further metabolized by the gut microbiota into smaller phenolic metabolites with increased bioavailability. Once in circulation, these phenolic metabolites can be transported to peripheral tissues, where they may accumulate either in the free form or as conjugates (12). The presence of phenolic-derived metabolites within muscle tissue can exert several functional effects on meat quality. Their antioxidant activity helps scavenge reactive oxygen species (ROS) and inhibit lipid peroxidation, thereby reducing the formation of secondary oxidation products. This mechanism contributes to improved oxidative stability of pork during storage, preserves color stability, and protects polyunsaturated fatty acids (PUFAs) from oxidative degradation (13). The Italian agri-food sector generates a substantial quantity of by-products, among which hazelnut skin (HS) is particularly noteworthy. Italy is the world’s second-largest producer of hazelnuts, accounting for approximately 20% of global production and 15% of exports. Among producing countries, Italy also records the highest per capita annual consumption of hazelnuts, calculated at 0.520 kg per person. It is estimated that 90% of the hazelnuts produced in Italy are industrially processed, with the remaining 10% consumed fresh (14, 15). Based on an average annual hazelnut production of approximately 110,000 tons, this corresponds to the generation of nearly 3,000 tons of HS per year (16). Due to their compositional characteristics and nutritional value, this by-product is well recognized and widely studied by the scientific community (17–22). These properties are largely attributed to their high content of macronutrients such as fiber and unsaturated fatty acids, as well as phenolic compounds with potent antioxidant activity. Previous research on dairy cows (23) and dairy sheep (24) has demonstrated that raw HS can partially replace conventional concentrated feeds without negatively affecting animal performance. Additionally, in sheep, dietary inclusion of HS has been associated with improved cheese sensory (24) and reduced lipid oxidation in meat (25). These benefits suggest that HS may also be valuable in monogastric nutrition, particularly in pigs. However, no studies have investigated the inclusion of this by-product in pig diets. The present study aimed to characterize HS and evaluate its incorporation into the finishing diet of heavy pigs in two forms: raw HS and its green polyphenolic extract (HSE). The objective was to assess their effects on growth performance, carcass traits, meat quality, and oxidative stability in gilts and barrows heavy pigs.

## 2 Materials and methods

### 2.1 Animal diets

Due to a lack of research on the use of HS in pig diets, its high crude fiber content, and findings by Tunçil (26) showing that over 96% of HS fiber is water-insoluble, comprising roughly 55% lignin and 45% fiber polysaccharides, to prevent any worsening of the animals’ performance, a minimal inclusion level was selected for the feed. The inclusion level was therefore determined based on the total polyphenol content of HS and HSE, expressed in mg of gallic acid equivalent (GAE)/g of sample. Drawing from the scientific literature on polyphenol supplementation in pig diets using by-products such as grape seed extract, bearberry (27), and apple (28), typically ranging from 400 to 850 mg GAE/kg of feed, the inclusion was adjusted to provide a total dietary polyphenol concentration of approximately 800–850 mg GAE/kg of feed.

Three types of diets were formulated: a maize-barley-soya bean meals basal diet (control diet, C), and two experimental formulations. In one, 0.6% of ground HS (particle size 2.5 mm), providing 843 mg GAE/kg feed, replaced 0.6% of wheat bran (HSD diet). In the other, the basal diet was supplemented with 0.1% of HSE, providing 837 mg GAE/kg feed (HSED diet). All three diets were isoproteic and isoenergetic and were formulated to meet nutritional requirements (29). The main characteristics of the diets are reported in Table 1. The dietary composition was analyzed in triplicate using an NIR FOSS 5000 spectroscope (Hilleroed, Denmark) at a laboratory specializing in animal feed analysis. Fatty acid composition (based on five samples per type of feed) and phenolic compound profiles (based on three samples per type of feed) were determined using the same methodologies previously described for HS and HSE in Sections 2.1.2 and 2.1.5, respectively. In addition, lipid hydroperoxides were determined according to the FOX assay protocol described by Cattivelli et al. (30).

**Table 1:** Ingredients (%), proximate composition (%, as fed basis), fatty acid composition (% of total fatty acids), phenolic compounds (mg/kg of feed), and lipid hydroperoxides (mEq H₂O₂/kg) of the diets.

Ingredients		C	HSD	HSED
Maize	%	50.00	50.00	50.00
Barley meal	%	20.00	20.00	20.00
Soybean meal	%	10.30	10.30	10.30
Wheat bran	%	10.00	9.40	10.00
Wheat middling	%	6.00	6.00	6.00
Calcium carbonate	%	1.30	1.30	1.30
Animal fat	%	1.00	1.00	1.00
HS	%	—	0.60	—
HSE	%	—	—	+0.10
Dicalcium phosphate dihydrate	%	0.50	0.50	0.50
Sodium chloride	%	0.50	0.50	0.50
Pre-mix1^1^	%	0.20	0.20	0.20
L-Lysine	%	0.20	0.20	0.20
Analysed composition (on fed basis)^2^				
Dry Matter	%	89.01	89.22	89.02
Crude protein	%	13.27	13.95	13.38
Crude fat	%	4.23	3.92	3.95
Crude fiber	%	4.13	3.53	4.03
Ashes	%	5.11	4.68	5.02
Calculated nutrients composition (on fed basis)^3^				
Digestible energy (DE)	MJ/kg	13.75	13.80	13.75
Calcium	%	0.71	0.71	0.71
Phosphorus	%	0.49	0.48	0.49
Digestible phosphorus	%	0.24	0.23	0.24
Lysine	%	0.76	0.76	0.76
Digestible lysine	%	0.66	0.66	0.66
Fatty acid composition (% of total FAs)				
Total saturated (SFA)		24.53	21.98	22.83
Total monounsaturated (MUFA)		32.64	30.08	28.11
Total polyunsaturated (PUFA)		42.83	47.94	49.07
Lipid hydroperoxides (mEq H₂O₂/kg)^4^		0.33	0.20	0.22
Phenolic compound (mg/kg feed)				
Total phenolic acids		0.37	38.41	38.96
Total flavonoids		0.96	88.64	104.63
Total phenolic compounds		1.33	127.05	143.59

HS, hazelnut skin; HSE, hazelnut skin extract; C, control group; HSD, experimental HS dietary group; HSED, experimental HSE dietary group; n.d., not detected. ^1^Providing the following nutrients (per kg diet as-fed): Vitamin A 5,200 IU; Vitamin D3 1,200 IU; Vitamin E (α-tocopheryl acetate) 16 mg; Vitamin K 1 mg; Vitamin B1 1.6 mg; Vitamin B5 8 mg; Vitamin B6 1.6 mg; Niacin 20 mg; Biotin 0.08 mg; Betaine 117 mg; Cu 14 mg; Fe 160 mg; Mn 48 mg; I 1.19 mg; Zn 60 mg; Se 0.24 mg; L-Lysine monohydrochloride 1,560 mg. ^2^NIR (Near infrared spectroscopy). ^3^Sauvant et al. (80). ^4^FOX assay (30).

SFA = Σ (C10:0, C14:0, C16:0, C17:0, C18:0, C20:0).

MUFA = Σ (C16:1, C17:1, C18:1, C20:1, C22:1).

PUFA = Σ (C18:2, C8:3n-3, C18:6n-6, C20:2n-6, C20:3n-3, C20:4n-6, C20:5n-3, C22:2n-6, C22:5n-3, C22:6n-3).

### 2.2 Animals, experimental design, and sample collection

All experimental procedures conducted in this study adhered to the guidelines set forth by the European Council Directive 2010/63/EU for the protection of animals used for scientific research, complied with Italian Legislative Decree No.26 of March 4, 2014, Article 2, Point F, and were approved by Ethics Committee for Animal Experimentation (OPBA) of the University of Modena and Reggio Emilia on May 11, 2022 (Prot. n. 212, Rep. n. 9/2025). The study involved 72 Italian Large White x (Italian Landrace x Italian Large White) pigs (Topigs Norsvin Italy) (average of live body weight (LBW): 113.9 ± 11.0 kg), intended for Protected Designation of Origin (PDO) Italian heavy pig production. The animals were balanced for gender (36 barrows and 36 gilts) and LBW and were evenly allotted into nine concrete-floored pens (10 m^2^ each), with eight pigs per pen. Pigs were randomly assigned to one of the following three dietary treatments for 101 days prior to slaughter, reaching a final average LBW of 175.8 ± 14.6 kg: (i) control group (C); (ii) HSD dietary group; (iii) HSED dietary group. Water was provided *ad libitum* through a nipple drinker system, and feed was also supplied *ad libitum* throughout the experimental period. The farm was located in the province of Modena, Italy (44°26′13″N, 10°41′27″E), at an altitude of 557 m above sea level. During the experimental period, the average environmental conditions were: temperature 16.0 °C ± 5.3 °C, relative humidity (RH) 70.3 ± 13.2%, and wind speed 5.9 ± 2.4 km/h. During the trial, the residual feed in each pen was weighed weekly, and pigs were individually weighed at three-time points: at the start, after 54 days, and at slaughter. Average daily gain (ADG), average daily feed intake (ADFI), and feed conversion ratio (FCR) were calculated (ADFI and FCR as the average value of each pen).

At the end of the trial, a subsample of 36 pigs (12 per treatment), balanced by pen and gender, was randomly selected and after an overnight fast, following the Council Regulation (EC) No. 1/2005 on the protection of animals during transport, the pigs were transported for 2 h to a commercial abattoir 160 kilometers away from the farm. There, they were electrically stunned and bled, following Council Regulation (EC) No. 1099/2009 on the protection of animals at the time of slaughter. All slaughter procedures were supervised by the Veterinary Service of the Italian Ministry of Health. After slaughter, the hot carcass weight of each animal was recorded. At the last rib level, on the split line of the carcass into two halves, backfat thickness was measured using a caliper. Subsequently, the carcasses were dissected into primal cuts, and each lean (thigh, loin, neck, and shoulder) and adipose cut (backfat, belly, jowl, and perirenal fat), along with the head, was weighed to determine its incidence on the hot carcass weight. During carcass dissection, the *longissimus thoracis* (LT) muscle was excised from the left side between the 4/5th and the last thoracic vertebrae for subsequent analyses. In addition, a backfat (BF) tissue sample was collected at the level of the last rib for fatty acid (FA) analysis. The entire LT and BF samples were transported in a refrigerated box to the laboratory of the Department and stored at 4 °C ± 1 °C until 24 h *postmortem* (p.m.). Each LT muscle was then sliced into five subsamples (~2.5 cm thick). Three of these, randomly selected, were used to assess pH, color, cooking loss, and lipid oxidative stability (LOS) at 24 h p.m., and after 3 and 7 days of refrigerated storage at 4 °C ± 1 °C. These samples were packed in resealable polypropylene containers without modifications in atmospheric gas concentration. LOS was measured both before and after cooking. The fourth LT subsample was used to measure drip loss, while the fifth LT subsample and the BF samples were vacuum-packed (Elegen, Reggio Emilia, Italy) and stored at −20 °C until the subsequent chemical analyses.

### 2.3 HS and HSE characterization

HS, derived from *Corylus Avellana* L., is an agro-food by-product obtained during the conventional industrial roasting process of hazelnuts. The HS used in this study was supplied by Regardia (Marene, Cuneo, Italy). HSE, a polyphenol-rich green extract derived from the same HS, was provided by the University of Turin and obtained through a subcritical fluid extraction technique, as described by Capaldi et al. (31). For chemical analyses, HS was ground using a Moulinex DPA 141 household mixer (Moulinex Italy) and sieved through a 500-micron mesh. All analyses on HS and HSE were performed in triplicate, and results were expressed as mean ± standard deviation (SD).

#### 2.3.1 Proximate composition

The chemical composition of HS and HSE was determined according to the Association of Official Analytical Chemists (32) procedures, and the results were expressed on wet basis.

#### 2.3.2 Fatty acid profile

The total lipids from HS, HSE, feed, and animal tissues were extracted following the Folch et al. (33) method. According to Zappaterra et al. (34), 50 mg of lipid extract was mixed with 2 mL of hexane and methylated by adding 200 μL of a 2 N methanolic potassium hydroxide solution (KOH from Carlo Erba, Milan, Italy; methanol from ITW Reagents, Barcelona, Spain). Fatty acids analyses were performed using a TRACE™GC Ultra (Thermo Electron Corporation, Rodano, Milano, Italy) equipped with a Flame Ionization Detector, a PVT injector and a TR-FAME Column (30 m length, 0.25 mm internal diameter, 0.2 μm film thickness) supplied by Thermo Scientific (Rodano, Milano, Italy). One μL of the methylated esters sample was injected into the GC with a split flow rate of 10 mL/min, operating at a constant flow of 1 mL/min of helium as a carrier gas. Both the detector and injector were maintained at 240 °C. After 2 min, the oven temperature was increased at a rate of 4 °C per min from 140 °C to 250 °C and then maintained for 5 min. The Chrom-card software (version 2.3.3, Thermo Electron Corporation, Rodano, Milano, Italy) was used to record, identify, and integrate the peaks of the fatty acid methyl esters (FAMEs). To identify the retention times of the FAMEs, a solution of standard FAMEs mix with known concentrations was used (Supelco 37) Component FAME mix, PUFA standard n.2, Animal Source (Supelco, Bellafonte, PA, USA), and individual FAMEs standard (Larodan, Fine Chemicals AB, Malmö, Sweden). The quantity of each FAME was expressed as the relative percentage of the total FAMEs content, using the normalized and correct area method.

#### 2.3.3 Extraction of phenolic compounds and total phenolic content (TPC) determination

The HS required the extraction of free phenolic compounds before performing assays for total phenolic content and antioxidant activity, whereas the HSE was directly diluted in water and used for the assays. The extraction procedure for free phenolic compounds from HS was carried out according to the method described by D’Ambra et al. (35) with some modifications. Briefly, 2.5 g of this by-product was homogenized with 12.5 mL of a methanol/water/formic acid solution (in a ratio of 70:28:2, v/v/v) using an Ultra-Turrax homogenizer (IKA, Germany) for 1 min. The resulting suspension was incubated at 37 °C for 30 min and then centrifuged at 6000 rpm for 15 min at 4 °C using a Remi Elektrotechnik LTD centrifuge (model NEYA 16R, Mumbai, India). The supernatant was collected, and the pellet was resuspended in 12.5 mL of fresh solution. This extraction step was repeated three times to completely extract the phenolic compounds from the initial 2.5 g sample. The polyphenol-rich extracts were stored at 0 °C–4 °C until further analysis.

The total phenolic content of HS and HSE was determined using the Folin–Ciocalteu assay (36) with some modifications. Briefly, 1,975 μL of distilled water was mixed with 25 μL of the extracted sample and 125 μL of Folin reagent (concentration 1.8–2.2 mol/L). After 1 min, 375 μL of a 20% Na_2_CO_3_ solution was added, and the mixture was incubated in the dark for 2 h. Following incubation, absorbance was measured at 765 nm using a Jasco UV/VIS spectrophotometer (model V550, Tokyo, Japan). Gallic acid was used to generate the calibration curve, and results were expressed as milligrams of gallic acid equivalents per gram of sample (mg GAE/g).

#### 2.3.4 ABTS, FRAP, and DPPH assays

The antioxidant capacity of HS and HSE was assessed using three different assays. Firstly, with the ABTS assay, following the protocol described by Re et al. (37). This method employs the chromogen reagent 2,2-azinobis-(3-ethylbenzothiazoline-6-sulfonic acid) (ABTS, AppliChem GmbH) to evaluate antioxidant activity. The assay was conducted by observing the decrease in absorbance at 734 nm of the ABTS• + radical cation in the presence of antioxidants. To generate the ABTS• + radical cation, a 7 mM aqueous solution of ABTS was mixed with 2.45 mM potassium persulfate and incubated in the dark overnight to allow the reaction to occur. The resulting ABTS• + solution was then diluted with methanol to achieve an initial absorbance value (*A_0_*) of 0.705 ± 0.005 at 734 nm. For the assay, 100 μL of the diluted sample was mixed with 1,400 μL of the ABTS• + solution and incubated at 20 °C for 15 min in the dark. The final absorbance at 734 nm (*A_f_*) was recorded, and the percentage of radical scavenging (*S%*) was calculated using the following equation:


$$S\%=\frac{\left(A_{0}-A_{f}\right)}{A_{0}}\times 100$$

Where *A_0_* represents the initial absorbance (control), and *A_f_* is the absorbance after reaction with the sample. Trolox (6-hydroxy 2,5,6,7-tetramethyl chroman-2-carboxyl acid) was used as the standard, and the ABTS scavenging capacity was quantified as mmol Trolox equivalents per gram of by-product, based on a calibration curve generated with Trolox concentrations ranging from 50 to 500 mmol/L under identical assay conditions.

Subsequently, the ferric reducing/antioxidant power (FRAP) of HS and HSE was determined according to the method described by Benzie and Strain (38). This assay is based on the reduction of the ferric (Fe^3+^)–2,4,6-tripyridyl-s-triazine (TPTZ) complex to its ferrous (Fe^2+^) form under acidic conditions. For the assay, 3 mL of freshly prepared FRAP reagent (20 mM ferric chloride solution, 10 mM TPTZ solution, and 0.3 M acetate buffer at pH 3.6) was mixed with 100 μL of the sample. After incubation at room temperature for 6 min, the absorbance was measured at 593. Results were expressed as μmol of FeSO*_4_* equivalents per gram of sample.

Finally, the antioxidant activity of HS and HSE was also evaluated using the DPPH (2,2-diphenyl-1-picrylhydrazyl) assay, following the procedure described by Helal et al. (39). A 0.1 mM DPPH solution was prepared in methanol and allowed to stabilize in the dark for 30 min. For the assay, 200 μL of the sample was mixed with 2 mL of the DPPH solution and incubated in a shaker in the dark for 30 min. The absorbance of the reaction mixture was then measured at 517 nm using a UV–visible spectrophotometer, with a blank containing no sample used as a reference. Antioxidant activity was calculated after correcting the sample blank absorbance and expressed as milligrams of Vitamin C equivalent per gram of sample.

#### 2.3.5 Identification and quantification of phenolic compounds by high-resolution mass spectrometry (UHPLC/MS)

The phenolic compound profiles of HS, HSE, and feeds were determined as reported by Cattivelli et al. (40). Prior to injection into the high-resolution mass spectrometer, phenolic compounds were extracted from the samples following the protocol outlined in Section 2.1.3 of the cited paper. The phenolic compounds were first separated using a C18 column (Acquity UPLC HSS C18 Reversed phase, 2.1 × 100 mm, 1.8 μm particle size, Waters, Milan, Italy) on a UHPLC Ultimate 3000 module system (Thermo Fisher Scientific, San Jose, CA, USA) and subsequently analyzed using a Q Exactive Hybrid Quadrupole-Orbitrap Mass Spectrometer (Thermo Fisher Scientific, San Jose, CA, USA). Chromatographic separation and mass spectrometry parameters were applied as described by Martini et al. (41). Quantification of phenolic compounds was performed using external calibration curves prepared with the available standard compounds.

### 2.4 Meat quality analysis

The pH value of each LT subsample was measured at 24 h p.m. and after 3 and 7 days of refrigerated storage using a portable Crison pH meter equipped with a Xerolite electrode (Crison Instruments, Alella, Spain), calibrated with solutions of known pH (4 and 7) and equipped with automatic temperature compensation. At the same time points, instrumental color measurements were performed using a Minolta CM-600d spectrophotometer (Konica Minolta Holdings, Inc., Osaka, Japan) equipped with an 8 mm aperture, a D65 illuminant, and a 10° standard observer. Following calibration with a standard white plate, three measurements were taken at different locations on each subsample, and the average values were recorded. Color results were expressed according to the CIE L*a*b* color space: L*- “lightness,” a*- “redness,” and b*- “yellowness.” In addition, color indices such as Chroma (C*) and Hue angle (H*) were calculated using the following equations:


$$C*=\sqrt[{2}]{a{*}^{2}+b{*}^{2.}}$$

H∗=arctangent(b∗/a∗)

Overall color changes in the samples (∆E_₂₄h₃__d_ and ∆E_₂₄h–₇__d_) were calculated as ∆E = (∆*L^2^* + ∆*a*^2^ + ∆*b*^2^)^1^´^2^, where ∆*L*, ∆*a*, and ∆*b* represent the differences in L*, a*, and b* values between 24 h p.m. and 3 or 7 days of storage, respectively.

Drip loss was assessed on LT samples starting at 24 h p.m., following the method of Honikel (42), with slight modifications. Briefly, a fresh LT slice, approximately 2.5 cm thick and weighing about 100 g, was weighed, placed in an inflated plastic bag ensuring no contact between the sample and the bag walls, and stored at 4 °C ± 1 °C for 48 h. Drip loss was calculated as the percentage difference between the initial and final weights.

Cooking loss was evaluated at 24 h p.m., and after 3 and 7 days of refrigerated storage on a 4×4 cm LT sample. Each sample was weighed before and after cooking on a household double-sided electric grill (Bosch, Germany) set at 80 °C for 3 min until the internal temperature reached 77.5 °C ± 8.5 °C. Measurements were conducted in triplicate. Cooking loss (%) was calculated as described by D’Ambra et al. (35):


$$\text{Cooking loss}\;(\%)=\frac{\text{weight}\;\mathrm{raw}\;\text{meat}-\text{weight cooked meat}}{\text{weight}\;\mathrm{raw}\;\text{meat}}\times 100$$

### 2.5 Oxidative stability of meat

Lipid oxidation was evaluated in raw and cooked LT samples at 24 h p.m. and after 3 and 7 days of refrigerated storage, using the method described by Siu and Draper (43) through the measurement of 2-thiobarbituric acid reactive substances (TBARS), as previously detailed by D’Ambra et al. (35). TBARS were expressed as mg of malondialdehyde (MDA) per kg of meat using 1,1,3,3 tetraethoxypropane (TEP, Sigma-Aldrich, Milan, Italy) as a standard.

### 2.6 Proximate composition and fatty acid profile

The chemical composition of LT samples was analyzed according to the Association of Official Analytical Chemists (32) methods, and the results were expressed on wet basis. The fatty acid profile of LT and BF samples was determined as previously described in Section 2.1.2.

The iodine value (IV) of BF samples was calculated based on their fatty acid composition, using the equations proposed by Lo Fiego et al. (44):


$$\begin{array}{l} \mathrm{IV}=85.703+[C14:0]\times 2.740-[C16:0]\times 1.085-\\ {}[C18:0]\times 0.710+[C18:2n-6]\times 0.986 \end{array}$$

Moreover, the atherogenic index (AI) and thrombogenic index (TI) of LT and BF samples were calculated according to the method proposed by Ulbricht and Southgate (45). AI was defined as:


$$\mathrm{AI}=\frac{C12:0+(4\times C14:0)+C16:0}{\sum \text{MUFA}+\sum n-6\text{PUFA}+\sum n-3\text{PUFA}}$$

and TI as:


TI=C14:0+C16:0+C18:0(0.5×∑MUFA)+(0.5×∑n−6PUFA)+(∑n−3PUFA∑n−6PUFA)

### 2.7 Cholesterol content

The concentration of cholesterol in LT was determined according to Bertolín et al. (46), with the following adaptations. Briefly, 2.5 g of the sample was mixed with 7.5 mL of 10% potassium hydroxide in 1:1 ethanol:water, along with 0.2 g of ascorbic acid, and then incubated on an orbital shaker overnight in the dark. Subsequently, 5 mL of 9:1 hexane:ethyl acetate was added. After the centrifugation of the samples, the supernatant was collected. This operation was repeated twice. Supernatants were dried under nitrogen flow, and residues were dissolved in 1 mL of methanol. Cholesterol quantification was performed using UHPLC as detailed in Natalello et al. (47), and results were expressed as mg/100 g of muscle tissue.

### 2.8 Statistical analysis

The data from HS and HSE characterization were reported as mean ± SD of three samples analyzed in triplicate. The data from the animal trial (live performance, carcass traits, meat quality traits within each storage time, and lipid composition) were subjected to statistical analysis using a Linear Mixed Model procedure of SAS, PDIFF option using T for multiple comparison adjustment (SAS Institute Inc., Cary, NC, USA). The statistical model included dietary treatment (C, HSD, and HSED), gender (gilts and barrows), and their interactions as fixed effects, and pen as a random effect.


$$Y_{\mathrm{ijk}}=\mu +\alpha_{i}+\beta_{j}+{\left(\alpha \beta \right)}_{\mathrm{ij}}+p_{k}+\varepsilon_{\mathrm{ijk}}$$

Where:

*μ*: overall mean

α_i_: fixed effect of diet (C, HSD, HSED)

β_J_: fixed effect of gender (gilts, barrows)

(αβ)_ij_: diet × gender interaction

p_k_: random effect of pen

ε_ijk_: residual error

The interaction of dietary treatments x gender was not found to be significant for any examined traits (*p* > 0.05) and therefore was not presented in the tables. Moreover, hot carcass weight, carcass yield, and backfat thickness were covariates for slaughter LBW. For average daily feed intake (ADFI) and feed conversion ratio (FCR) calculation, the average data of each pen containing 8 pigs was considered as starting data (experimental unit). For all other parameters measured in live animals, carcasses, and tissues, the experimental unit was the individual pig. Significant differences were declared at least *p* < 0.05, unless otherwise specified.

## 3 Results

### 3.1 HS and HSE extract characterization

Table 2 shows the data characterizing the HS and its green extract (HSE).

**Table 2:** Proximate composition, fatty acid composition, total phenolic content (TPC), antioxidant activity (ABTS, FRAP, and DPPH assay), and phenolic compounds of HS and HSE (Mean ± SD of 3 samples analyzed in triplicate).

Parameter	HS (*n* = 3)	HSE (*n* = 3)
Moisture %	7.04 ± 0.04	6.99 ± 0.15
Crude lipids %	25.00 ± 0.001	n.d.
Crude protein %	9.36 ± 1.25	n.d.
Crude fiber %	16.97 ± 5.56	n.d.
Ashes %	1.95 ± 0.00	0.06 ± 0.00
Fatty acid (FA) composition (% of total FAs)		
C16:0 (palmitic)	5.71 ± 0.07	—
C16:1 (palmitoleic)	0.15 ± 0.01	—
C18:0 (stearic)	2.14 ± 0.02	—
C18:1n-9 (oleic)	75.86 ± 0.12	—
C18:2n-6 (linoleic)	15.03 ± 0.02	—
C18:3n-3 (linolenic)	0.17 ± 0.00	—
C20:0 (eicosanoic)	0.13 ± 0.00	—
C20:1 (eicosenoic)	0.17 ± 0.00	—
C22:5–3 (docosapentaenoic)	0.64 ± 0.04	—
Total n-6 PUFA	15.03 ± 0.02	—
Total n-3 PUFA	0.82 ± 0.05	—
Total saturated fatty acids (SFA)	7.98 ± 0.07	—
Total monounsaturated (MUFA)	76.17 ± 0.04	—
Total polyunsaturated fatty acids (PUFA)	15.85 ± 0.03	—
TPC (mg GAE/g)	140.44 ± 16.36	837.00 ± 0.02
ABTS (mmol Trolox eq/g)	1068.78 ± 12.22	2472.73 ± 154.3
FRAP (μmol FeSO_4_/g)	540.36 ± 8.06	2951.37 ± 13.60
DPPH (mg Vitamin C/g)	262.69 ± 5.96	793.10 ± 32.20
High-resolution mass spectrometry		
Total phenolic acids (mg/100 g)	17.02 ± 0.03	108.52 ± 0.58
Total flavonoids (mg/100 g)	487.39 ± 0.56	1012.56 ± 2.69
Total phenolic compounds (mg/100 g)	504.41 ± 1.01	1121.08 ± 4.76

HS, hazelnut skin; HSE, hazelnut skin extract; TPC, total polyphenolic content, detected by Folin–Ciocalteu assay; GAE, gallic acid equivalent; ABTS, 2, 2 -azinobis-(3-ethylbenzothiazoline-6-sulfonic); FRAP, ferric reducing/antioxidant power; DPPH, 2,2-diphenyl-1-picrylhydrazy; n.d., not detected.

Both products exhibited a low moisture content (7.0%), making them suitable for long-term storage, an essential requirement for feed and food additives. HS was characterized by a substantial fiber content (16.97%) and a satisfactory protein content (9.36%). Its lipid fraction (25.00%) was primarily composed of oleic acid (75.86%), followed by linoleic acid (15.03%) and palmitic acid (5.71%). As expected, proteins, lipids, and fiber were absent in HSE.

The content of phenolic compounds (TPC, mg GAE/g) was approximately six times higher in the HSE, indicating a high extraction yield. However, antioxidant activity, as measured by ABTS, FRAP, and DPPH assays, while higher in the HSE, does not reflect the same ratio observed in the total phenolic content (TPC).

In terms of phenolic compound classes, the two products were qualitatively similar, emphasizing the extraction effectiveness of the method, with flavonoids representing the predominant class, accounting for 96.6% in HS and 90.3% in HSE, followed by the phenolic acids class, which comprised 3.4 and 9.7% in HS and HSE, respectively. Within the flavonoid category, flavan-3-ols were the major constituents (81.8% in HS and 91.3% in HSE), followed by flavonols (18.2% and 8.7% in HS and HSE) (Data not reported in the table).

### 3.2 Feed composition

Diets including HS or HSE (Table 1) showed an increased phenolic compounds content (127.05 and 143.59 mg/kg of feed, respectively) compared to the C diet (1.33 mg/kg of feed). Furthermore, the diets supplemented with HS and HSE showed a higher content of polyunsaturated fatty acids and lower values of lipid hydroperoxide.

### 3.3 Growth performance and carcass traits

Table 3 shows the effect of dietary treatment and gender on live performance.

**Table 3:** Effect of dietary treatment and gender on live performance (Least square means and standard error (SEM) of the means).

Parameter	Dietary treatments				Gender		
	C	HSD	HSED	SEM	Gilts	Barrows	SEM
N. of pigs	24	24	24		36	36	
Initial LBW (kg)	114.6	114.5	112.6	2.24	111.8	116.0	1.83
ADG (kg)	0.59	0.64	0.61	0.02	0.61	0.62	0.02
ADFI^(1)^ (kg/day)	2.40	2.42	2.39	0.05	—	—	—
FCR^(1)^ (kg*kg^−1^)	4.05	3.81	3.92	0.12	—	—	—
Slaughter LBW (kg)	174.5	178.7	174.3	2.75	173.3	178.4	2.24

C, control group; HSD, experimental HS dietary group; HSED, experimental HSE dietary group.

LBW, live body weight; ADG, average daily gain; ADFI, average daily feed intake; FCR, feed conversion ratio; ^(1)^ mean values of each pen were considered as raw starting data.

*In vivo* performance was not influenced by either dietary treatment or gender (*p* > 0.05). Pigs that started the trial with a fairly balanced live body weight across dietary and gender groups showed, on average, similar daily feed intake and daily gain, with no significant differences observed in feed conversion ratio or slaughter weight. Nonetheless, pigs fed the HSD diet showed a slight tendency toward greater average daily gain (+0.05 kg), heavier slaughter weight (+4.2 kg), and a slightly improved feed conversion ratio (−0.24 kg*kg^−1^), compared with the C group.

The effects of dietary treatment and gender on carcass traits are reported in Table 4.

**Table 4:** Effect of dietary treatment and gender on carcass traits (Least square means and standard error (SEM) of the means).

Item	Dietary treatments				Gender		
	C	HSD	HSED	SEM	Gilts	Barrows	SEM
Hot carcass weight (kg)^#^	147.8	146.9	144.8	1.37	145.0	148.0	1.13
Hot carcass yield (%)^#^	81.59	81.06	80.04	0.76	80.05	81.74	0.63
Backfat thickness (mm)^#^	25.42	25.44	26.05	1.23	24.13^b^	27.15^a^	1.02
Lean cuts (%)^1^							
Thigh	26.35^B^	26.64^B^	27.71^A^	0.31	26.89	26.91	0.24
Loin	18.47	19.05	18.86	0.39	19.24^a^	18.34^b^	0.32
Neck	7.20	7.17	7.22	0.13	7.34^a^	7.06^b^	0.09
Shoulder	14.84	14.21	14.37	0.23	14.58	14.36	0.06
Total lean cuts	66.86^B^	67.07^B^	68.15^A^	0.73	68.05^a^	66.67^b^	0.59
Adipose cuts (%)^1^							
Backfat	4.46	4.37	3.74	0.27	3.92^b^	4.46^a^	0.21
Belly	13.21	13.41	13.08	0.35	13.27	13.19	0.26
Jowl	7.81	7.24	7.32	0.20	7.21^b^	7.70^a^	0.14
Perirenal fat	1.71	1.72	1.63	0.13	1.45^B^	1.92^A^	0.10
Total adipose cuts	27.19	26.73	25.76	0.73	25.85^b^	27.26^a^	0.57
Head (%)^1^	4.65	4.91	4.79	0.13	4.79	4.77	0.10

C, control group; HSD, experimental HS dietary group; HSED, experimental HSE dietary group.

^#^Covariate for slaughter LBW.

^1^as % of hot carcass weight.

Within each effect, different letters in the same line indicate statistically different means for *p* < 0.05^(a, b,c)^ or *p* < 0.01^(A, B, C)^.

Hot carcass weight, carcass yield, and backfat thickness, covariate for slaughter live weight, were not affected by either dietary treatment or gender. However, for the main commercial cuts, the HSED group had the highest percentage of lean cuts (*p* < 0.01), driven by a significantly higher thigh yield (*p* < 0.01) than in the C and HSD groups. No significant differences in adipose cuts were observed among dietary treatments.

Concerning gender, gilts exhibited a higher (*p* < 0.05) lean cuts percentage compared to barrows, particularly loin and neck (*p* < 0.05). Conversely, barrows had significantly higher (*p* < 0.05) percentage of adipose cuts, especially backfat, jowl (*p* < 0.05), and perirenal fat (*p* < 0.01).

### 3.4 Chemical and physical characteristics of *longissimus thoracis* muscle

Table 5 reports, within each time p.m. considered, the chemical and physical characteristics of LT.

**Table 5:** Effect of dietary treatment and gender on chemical and physical characteristics of *longissimus thoracis* muscle at 24 h postmortem and during refrigerated storage at 4 °C ± 1 °C for 3 and 7 days (Least square means and standard error (SEM) of the means).

Parameter	Dietary treatments				Gender		
	C	HSD	HSED	SEM	Gilts	Barrows	SEM
24 h postmortem							
pH	5.54^b^	5.64^a^	5.59^a,b^	0.03	5.59	5.59	0.02
L*	57.33	56.45	55.40	1.10	57.78^a^	55.00^b^	0.90
a*	1.52	1.69	1.71	0.23	1.65	1.62	0.19
b*	11.49^a,b^	11.11^a^	10.65^b^	0.32	11.21	10.90	0.26
Chroma (C*)	11.60	11.26	10.73	0.33	11.36	11.04	0.27
Hue angle (H*)	82.51	81.40	80.96	1.11	81.67	81.58	0.90
Moisture (%)	73.26	73.30	72.88	0.30	73.25	73.05	0.25
Ether extract (%)	2.25	1.62	2.30	0.40	1.87	2.24	0.35
Protein (%)	23.15	23.22	23.21	0.22	23.24	23.14	0.18
Drip loss (%) ^(1)^	4.33	3.87	4.05	0.56	4.40	3.76	0.49
Cooking loss (%)	28.15^d^	26.33^c,d^	24.84^e^	1.27	26.36	26.53	1.04
MDA (mg/kg) raw	0.172	0.135	0.154	0.03	0.168	0.138	0.02
MDA (mg/kg) cooked	0.389	0.338	0.320	0.04	0.371	0.327	0.03
3 days of refrigerated storage							
pH	5.57^b^	5.70^a^	5.60^a^	0.03	5.61	5.64	0.02
L*	59.26	58.22	57.52	0.93	58.23	58.43	0.76
a*	4.51	4.63	4.27	0.43	4.23	4.71	0.35
b*	13.44	13.17	12.71	0.40	12.89	13.32	0.33
Chroma (C*)	14.22	13.98	13.45	0.50	13.61	14.16	0.41
Hue angle (H*)	71.73	71.07	71.95	1.28	72.30	70.87	1.05
∆E 24h_3d	4.61	5.13	4.94	0.61	4.31	5.48	0.50
Cooking loss (%)	27.14	27.05	26.97	1.19	26.79	27.31	0.97
MDA (mg/kg) raw	0.183	0.164	0.170	0.02	0.178	0.166	0.02
MDA (mg/kg) cooked	0.326	0.321	0.289	0.04	0.314	0.310	0.03
7 days of refrigerated storage							
pH	5.53^b^	5.64^a^	5.59^a,b^	0.03	5.59	5.59	0.02
L*	59.79	59.30	58.60	1.03	59.72	58.74	0.85
a*	4.03	4.24	3.95	0.37	3.82	4.33	0.30
b*	13.26	13.20	12.87	0.36	13.01	13.22	0.29
Chroma (C*)	13.89	13.89	13.51	0.42	13.58	13.94	0.35
Hue angle (H*)	73.24	72.43	73.47	1.30	73.92	72.17	1.12
∆E 24h_7d	4.04	5.03	5.55	0.61	4.18^e^	5.57^d^	0.50
Cooking loss (%)	25.62	26.36	24.53	1.18	25.43	25.58	1.01
MDA (mg/kg) raw	0.223	0.209	0.217	0.02	0.209	0.224	0.01
MDA (mg/kg) cooked	0.390^d^	0.322^e^	0.338^de^	0.02	0.347	0.353	0.02

C, control group; HSD, experimental HS dietary group; HSED, experimental HSE dietary group; MDA, malondialdehyde.

^(1)^After 48 h of refrigerated storage (4°C).

Different letters in the same line indicate statistically different means for *p* < 0.05^(a,b,c)^ or *p* < 0.08^(d,e,f)^ within each storage time.

Overall, dietary treatment did not significantly affect most of the qualitative traits evaluated in LT, except for pH, which was higher in the treated groups compared to the control group. The HSD group consistently showed a significantly higher pH value (*p* < 0.05) with respect to the C group, whereas the HSED group differed from the C group (*p* < 0.05) only after 3 days of refrigerated storage. Additionally, at 24 h p.m., the HSED group exhibited a lower b* value (*p* < 0.05) than the HSD group and tended to have lower cooking loss values (*p* < 0.08) than the C group.

Gender did not significantly affect the qualitative traits of the LT, except for the L* value at 24 h p.m., which was lower (*p* < 0.05) in barrows, and ∆the E value at day 7 of storage, which was tendentially higher (*p* < 0.08) in the latter. The chemical composition of the LT (moisture, ether extract, and protein) was not influenced by either gender or dietary treatment. Table 5 also shows the malondialdehyde (MDA) content of raw and cooked samples during refrigerated storage. No significant differences (*p* > 0.05) were observed between the control and treated groups, except in cooked samples on day 7, where the HSD group tended to show a lower MDA concentration (*p* < 0.08) compared to the C group, while the HSED group displayed intermediate values.

**Figure 1:**
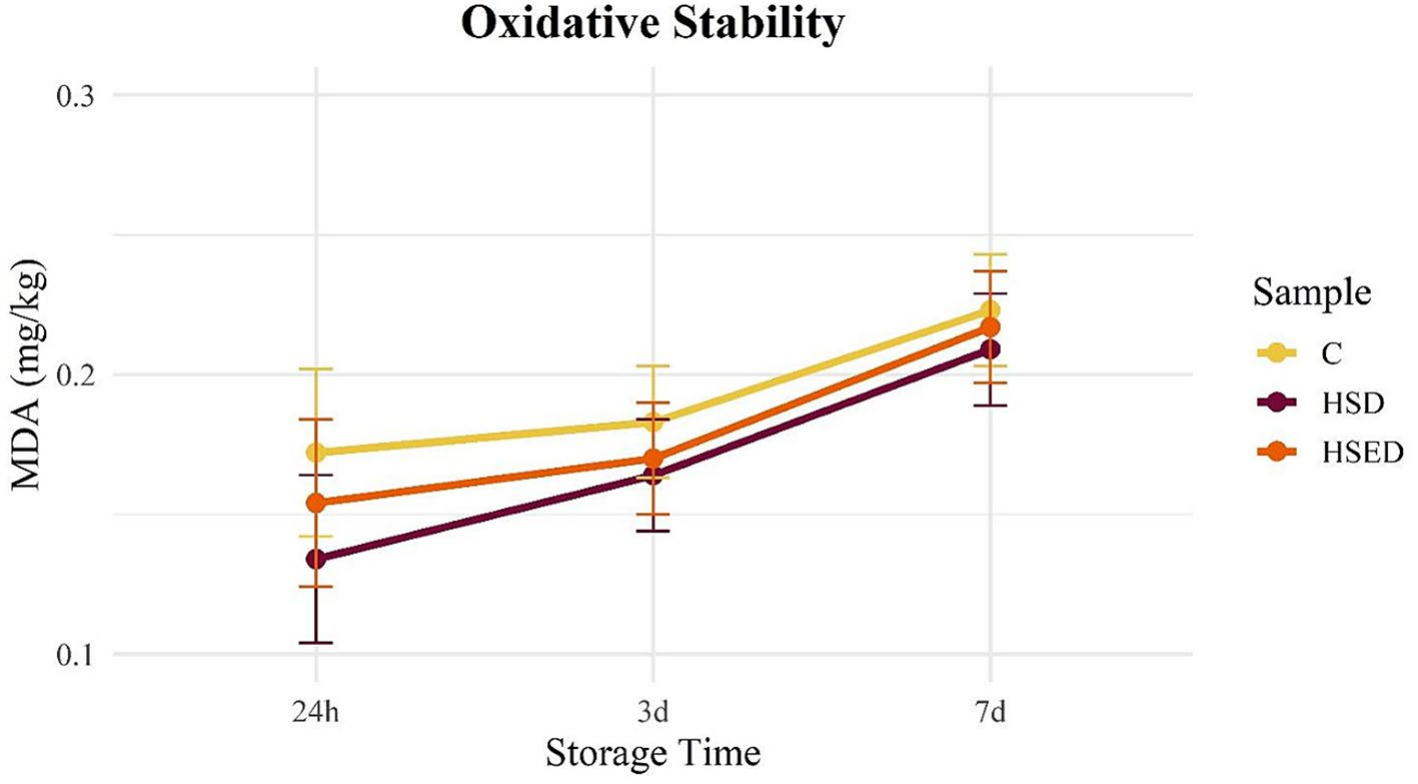
Variations of malondialdehyde (MDA mg/kg) content during refrigerated storage of raw *longissimus thoracis* muscle samples. C, control group; HSD, experimental bars represent the standard error of the means.

**Figure 2:**
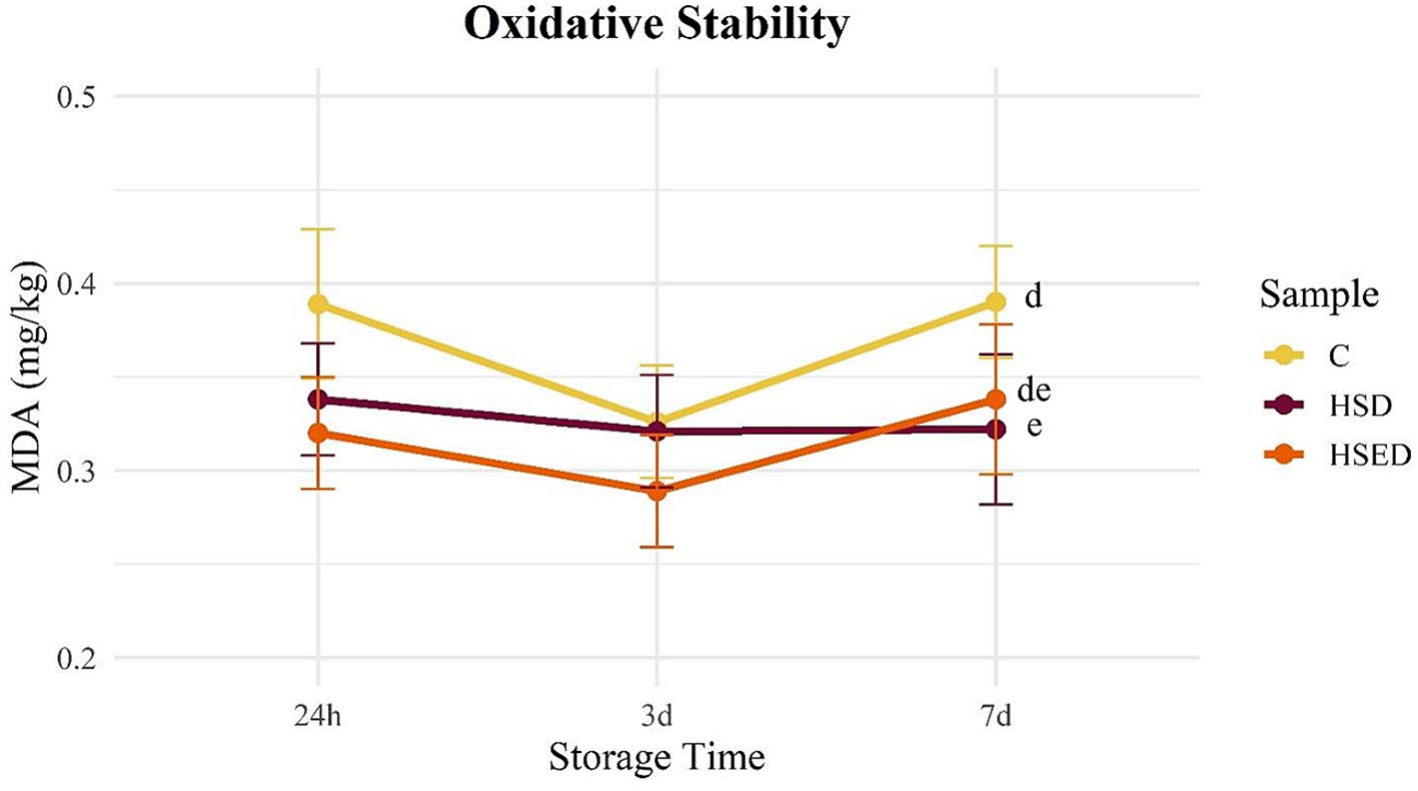
Variations of malondialdehyde (MDA mg/kg) content in cooked *longissimus thoracis* muscle samples after different storage times. C, Control group; HSD, experimental HS dietary group; HSED, experimental HSE dietary group. Different letters indicate different means for *p* < 0.08^(d,e)^ within storage time. Error bars represent the standard error of the means.

Figures 1, 2 illustrate the temporal trends in MDA content for raw (Figure 1) and cooked (Figure 2) meat samples during refrigerated storage. In raw samples, all three groups followed a similar increasing pattern over time. In cooked samples, however, the C and HSED groups exhibited a comparable trend; MDA levels in the HSD group remained relatively stable across the three sampling days. Although no statistical differences were detected between groups over storage time, the supplemented groups consistently showed numerically lower MDA values than the C group in both raw and cooked meat. MDA content was not influenced by gender (Table 5).

### 3.5 Fatty acid profile of *longissimus thoracis* muscle and backfat tissue

The fatty acid profile of *longissimus thoracis* muscle is reported in Table 6.

**Table 6:** Effect of dietary treatments and gender on fatty acid profile (% of total fatty acid) of *longissimus thoracis* muscle (Least square means and standard error (SEM) of the means).

Parameter	Dietary treatments				Gender		
	C	HSD	HSED	SEM	Gilts	Barrows	SEM
C10:0 (capric)	0.08^b^	0.12^a^	0.11^ab^	0.01	0.10	0.11	0.01
C12:0 (lauric)	0.09	0.09	0.09	0.00	0.09	0.09	0.00
C14:0 (myristic)	1.37	1.35	1.37	0.04	1.33	1.40	0.03
C16:0 (palmitic)	24.15	23.98	24.10	0.31	23.76	24.38	0.25
C17:0 (heptadecanoic)	0.10^b^	0.18^a^	0.17 ^a^	0.02	0.16	0.14	0.01
C18:0 (stearic)	12.38	12.40	11.83	0.24	12.40	12.01	0.19
C20:0 (eicosanoic)	0.13^a,b^	0.18^a^	0.11^b^	0.02	0.13	0.14	0.02
C16:1 (palmitoleic)	3.54	3.43	3.58	0.11	3.40	3.63	0.01
C17:1 (heptadecenoic)	0.18	0.18	0.22	0.02	0.21	0.18	0.02
C18:1n-7 (vaccenic)	4.05	3.98	4.12	0.21	4.23	3.86	0.17
C18:1n-9 (oleic)	39.61	38.36	39.66	0.77	37.99^B^	40.43^A^	0.63
C20:1 (eicosenoic)	0.67	0.65	0.65	0.02	0.62^b^	0.69 ^a^	0.02
C18:2n-6 (linoleic)	9.95	10.88	10.17	0.56	11.11^a^	9.55^b^	0.46
C18:3n-3 (α-linolenic)	0.30	0.31	0.30	0.01	0.31	0.30	0.01
C18:3n-6 (γ-linolenic)	0.06	0.05	0.05	0.00	0.06	0.05	0.00
C20:2n-6 (eicosadienoic)	0.32	0.29	0.33	0.02	0.31	0.31	0.02
C20:4n-6 (arachidonic)	2.54	2.84	2.58	0.36	3.07^a^	2.23^b^	0.29
C20:5n-3 (eicosapentaenoic)	0.06	0.06	0.06	0.01	0.06	0.05	0.01
C22:4n-6 (docosatetraenoic)	0.21^b^	0.39 ^a^	0.33^ab^	0.05	0.37^a^	0.25^b^	0.04
C22:5n-3 (docosapentaenoic)	0.02	0.03	0.02	0.01	0.03	0.02	0.00
C22:6n-3 (docosahexaenoic)	0.17 ^b^	0.26^a^	0.15 ^b^	0.03	0.23^a^	0.15^b^	0.02
Total saturated (SFA)	38.30	38.30	37.78	0.50	37.98	38.27	0.41
Total monounsaturated (MUFA)	48.06	46.59	48.23	0.71	46.45^B^	48.80^A^	0.58
Total polyunsaturated (PUFA)	13.64	15.11	13.99	0.96	15.57^a^	12.93^b^	0.79
Total n-6 PUFA	13.09	14.44	13.47	0.93	14.93^a^	12.40^b^	0.76
Total n-3 PUFA	0.55^b^	0.67^a^	0.53^b^	0.04	0.64^a^	0.52^b^	0.03
n-6/n-3 PUFA ratio	23.90^a,b^	21.57^b^	25.70^a^	0.95	23.55	23.90	0.77
Atherogenic index (AI)	0.48	0.48	0.48	0.01	0.47	0.49	0.01
Thrombogenic index (TI)	1.18	1.16	1.15	0.03	1.15	1.17	0.02

C, control group; HSD, experimental HS dietary group; HSE, experimental HSE dietary group.

AI = [C12:0 + (4× C14:0) + C16:0]/[n-6PUFA + n-3PUFA + MUFA] (45).

TI = [C14:0 + C16:0 + C18:0]/[(0.5× MUFA) + (0.5× n-6PUFA) + (n-3PUFA/n-6PUFA)] (45).

Within each effect, different letters in the same line indicate statistically different means for *p* < 0.05^(a,b,c)^ or *p* < 0.01^(A,B,C)^.

Overall, the dietary treatment did not affect in a relevant way the proportions of fatty acid classes in LT. However, the C group showed a lower percentage of capric acid (C10:0), heptadecanoic acid (C17:0), docosatetraenoic acid (C22:4n-6), and docosahexaenoic acid (C22:6n-3), as well as a reduced total n-3 PUFA content compared to the HSD group (*p* < 0.05).

The HSED group exhibited a significantly higher level of C17:0 acid compared to the C group (*p* < 0.05), but lower concentrations of eicosanoic acid (C20:0), C22:6n-3 acid, total n-3 PUFA, and a higher n-6/n-3 PUFA ratio than the HSD group (*p* < 0.05). Despite these differences, the n-6/n-3 PUFA ratio, atherogenic index, and thrombogenic index remained unaffected.

Regarding gender, barrows had a significantly higher (*p* < 0.01) content of monounsaturated fatty acids, particularly due to high levels of oleic acid (C18:1n-9) (*p* < 0.01) and eicosenoic acid (C20:1) (*p* < 0.05). In contrast, gilts exhibited a higher (*p* < 0.05) total PUFA content, including both n-3 and n-6 PUFA, due to higher percentages of linoleic (C18:2n-6), arachidonic (C20:4n-6), C22:4n-6, and C22:6n-3 acids (*p* < 0.05). Gender did not influence either the n-6/n-3 PUFA ratio or the atherogenic and thrombogenic indices.

Table 7 reports the fatty acid profile of backfat tissue.

**Table 7:** Effect of dietary treatments and gender on fatty acid profile (% of total fatty acid) of backfat tissue (Least square means and standard error (SEM) of the means).

Parameter	Dietary treatments				Gender		
	C	HSD	HSED	SEM	Gilts	Barrows	SEM
C10:0 (capric)	0.06	0.06	0.06	0.00	0.06	0.06	0.00
C12:0 (lauric)	0.10	0.10	0.09	0.00	0.10	0.10	0.00
C14:0 (myristic)	1.35^a^	1.32^a,b^	1.28^b^	0.02	1.30	1.34	0.02
C16:0 (palmitic)	24.68	24.46	24.33	0.27	24.35	24.64	0.22
C17:0 (heptadecanoic)	0.29	0.30	0.29	0.02	0.29	0.30	0.01
C18:0 (stearic)	13.85	14.31	14.01	0.24	14.30	13.81	0.19
C20:0 (eicosanoic)	0.30	0.29	0.27	0.02	0.29	0.28	0.01
C16:1 (palmitoleic)	1.79^a^	1.59^b^	1.62^b^	0.04	1.62	1.70	0.04
C17:1 (heptadecenoic)	0.23	0.23	0.25	0.01	0.24	0.24	0.01
C18:1n-7 (vaccenic)	2.45	2.33	2.31	0.20	2.24	2.48	0.16
C18:1n-9 (oleic)	37.18^a^	35.82^b^	36.37^a,b^	0.41	36.22	36.69	0.33
C20:1 (eicosenoic)	0.85	0.85	0.84	0.03	0.82	0.87	0.02
C18:2n-6 (linoleic)	14.95^b^	16.26^a^	16.22^a^	0.33	16.14	15.48	0.27
C18:3n-3 (α-linolenic)	0.74	0.75	0.77	0.02	0.76	0.75	0.01
C18:3n-6 (γ-linolenic)	0.07	0.06	0.06	0.01	0.06	0.06	0.01
C20:2n-6 (eicosadienoic)	0.70	0.74	0.74	0.02	0.72	0.73	0.01
C20:3n-3 (eicosatrienoic)	tr^c^	0.13^a^	0.07^b^	0.01	0.06	0.07	0.01
C20:4n-6 (arachidonic)	0.22	0.22	0.24	0.01	0.23	0.22	0.01
C20:5n-3 (eicosapentaenoic)	tr	tr	tr	0.00	tr	tr	0.00
C22:4n-6 (docosatetraenoic)	0.11	0.09	0.11	0.01	0.11	0.10	0.01
C22:5n-3 (docosapentaenoic)	tr	tr	tr	0.00	tr	tr	0.00
C22:6n-3 (docosahexaenoic)	0.06	0.07	0.07	0.01	0.07	0.07	0.00
Total saturated (SFA)	40.64	40.85	40.34	0.46	40.69	40.53	0.38
Total monounsaturated (MUFA)	42.51^a^	40.82^b^	41.38^b^	0.33	41.15	41.99	0.27
Total polyunsaturated (PUFA)	16.85^b^	18.33^a^	18.28^a^	0.37	18.16	17.48	0.30
Total n-6 PUFA	16.04^b^	17.38^a^	17.37^a^	0.35	17.27	16.60	0.29
Total n-3 PUFA	0.81^b^	0.95^a^	0.91^a^	0.02	0.89	0.89	0.02
n-6/n-3 PUFA ratio	19.88^a^	18.28^b^	19.34^a^	0.37	19.50	18.84	0.30
Atherogenic index (AI)	0.51	0.50	0.50	0.01	0.51	0.50	0.01
Thrombogenic index (TI)	1.26	1.25	1.23	0.02	1.25	1.25	0.02
Iodine value (IV)	67.52	68.50	68.85	0.64	68.60	68.08	0.52

C, control group; HSD, experimental HS dietary group; HSE, experimental HSE dietary group; tr, trace.

AI = [C12:0 + (4*C14:0) + C16:0]/[n-6PUFA + n-3PUFA + MUFA] (45).

TI = [C14:0 + C16:0 + C18:0]/[(0.5*MUFA) + (0.5*n-6PUFA) + (n-3PUFA/n-6PUFA)] (45).

IV = (85.703 + [C14:0] × 2.740 − [C16:0] × 1.085 − [C18:0] × 0.710 + [C18:2n-6] × 0.986) (44).

Within each effect, different letters in the same line indicate statistically different means for *p* < 0.05^(a,b,c)^.

In BF tissue, HSD and HSED groups exhibited lower (*p* < 0.05) levels of monounsaturated fatty acids compared to the C group, primarily due to reduced (*p* < 0.05) levels of palmitoleic acid (C16:1) and, in the case of the HSD group, also lower (*p* < 0.05) levels of oleic acid. Additionally, the treated groups showed a higher (*p* < 0.05) content of polyunsaturated fatty acids, mainly due to increased (*p* < 0.05) levels of linoleic acid, as well as a higher (*p* < 0.05) n-6 and n-3 PUFA contents compared to the C group. Regarding the n-6/n-3 PUFA ratio, the HSD group displayed a lower (*p* < 0.05) value than the other two groups. No significant differences were observed between the groups for the atherogenic index, thrombogenic index, and iodine value. Gender did not influence the fatty acid profile of backfat.

### 3.6 Cholesterol content of *longissimus thoracis* muscle

**Figure 3:**
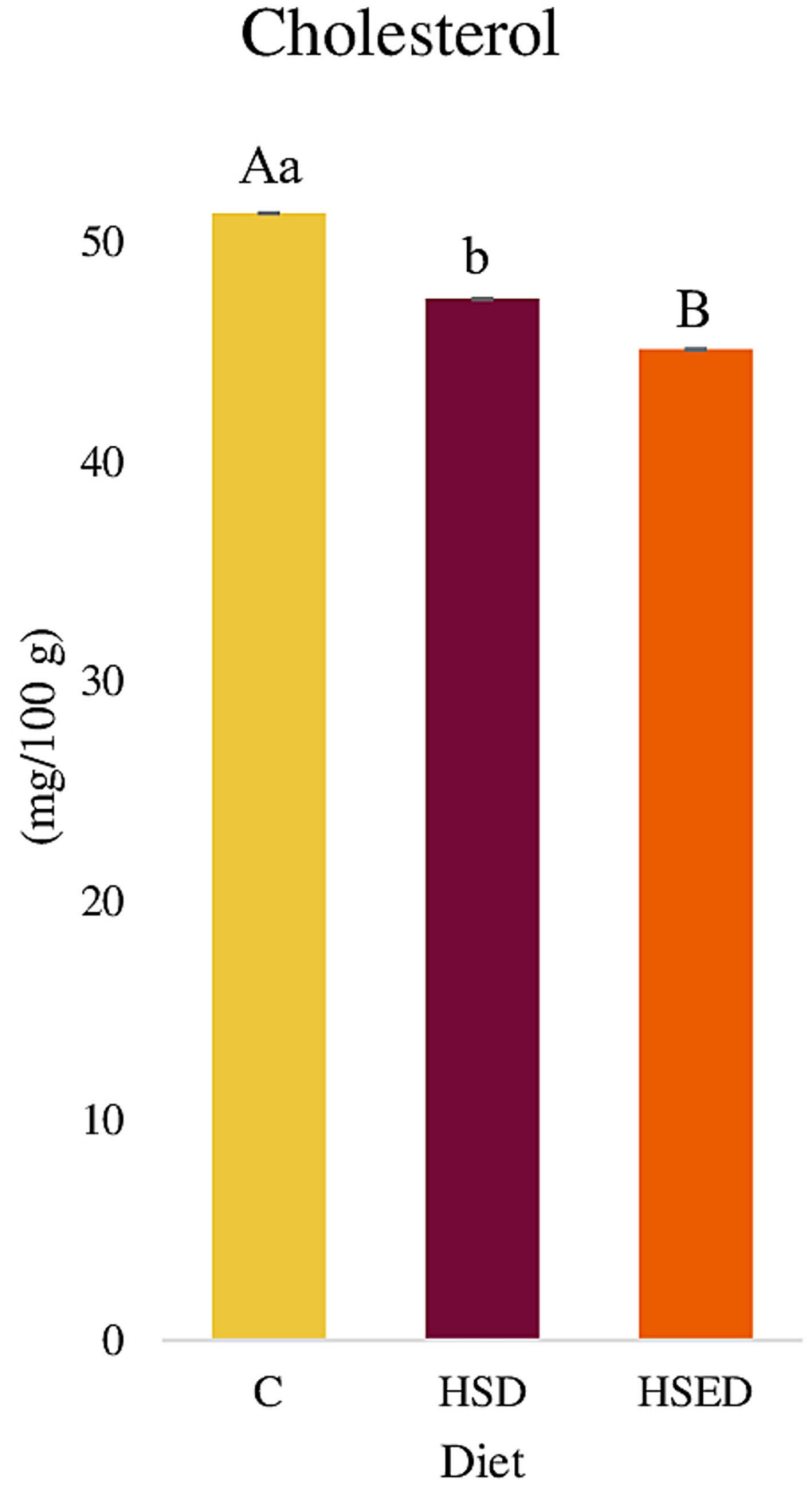
Cholesterol content in *longissimus thoracis* muscle (mg/100 g). C, Control group; HSD, experimental HS dietary group; HSED, experimental HSE dietary group. Different letters indicate different means within dietary group for *p* < 0.05^(a,b)^ or *p* < 0.01^(A,B)^. Error bars represent the standard error of the means.

Figure 3 shows the results relating to the cholesterol content of the LT muscle.

The control group exhibited the highest total cholesterol content in the LT muscle tissue (51.3 mg/100 g) with respect to the HSD group (47.4 mg/100 g, *p <* 0.05) and to the HSED group (45.1 mg/100 g, *p <* 0.01). No statistical differences (*p* > 0.05) were detected between HSD and HSED groups (Figure 3) and between genders (data not shown).

## 4 Discussion

The objective of this study was to evaluate the potential of incorporating hazelnut skin (HS) or its green phenolic extract (HSE) into finishing pig diets to maintain or improve feed efficiency, meat quality, and oxidative stability. The results showed that HS is characterized by a low moisture content (7%), a feature that facilitates its transportation, storage, and processing for use as an ingredient in animal feed. However, its relatively high fiber content (approximately 17%) may limit its inclusion at high levels in diets for monogastric animals such as pigs, as excessive dietary fiber may negatively affect nutrient digestibility. This limitation could potentially be mitigated by using its green polyphenolic extract (HSE), although the extraction process may increase production costs. HS is also characterized by a relatively high lipid content (approximately 25%), mainly composed of oleic (76%) and linoleic (15%) acids. In addition, it contains a moderate amount of protein (9.4%) and a high concentration of antioxidant compounds, particularly flavonoids (487 mg/100 g in HS and 1,013 mg/100 g in HSE) and phenolic acids (17 mg/100 g in HS and 109 mg/100 g in the extract). The high levels of phenolic compounds detected in both HS and HSE are consistent with the strong antioxidant activity measured using ABTS, FRAP, and DPPH assays. Similar results were reported by Muzolf-Panek et al. (48), who described a plant-based supplement containing approximately 150 mg of gallic acid equivalents (GAE) per gram, a value comparable to that observed in our HS (140.44 ± 16.4 mg GAE/g).

The inclusion of HS or HSE resulted in a higher concentration of phenolic compounds in the diets of the supplemented groups compared with the control group (127.05, 143.59, and 1.33 mg/kg feed in HSD, HSED, and C dietary groups, respectively). This increased phenolic content may have contributed to the lower lipid hydroperoxide levels and the higher PUFA content observed in the supplemented feeds. It is well established that lipid oxidation leads to a reduction in polyunsaturated fatty acids and an increase in saturated fatty acids and oxidation products (49, 50). Therefore, the antioxidant compounds present in HS and HSE may have contributed to preserving lipid quality in the feed.

Despite these compositional characteristics, the *in vivo* results showed that dietary treatments did not significantly affect growth performance, feed intake, or feed efficiency. This outcome may be explained by the fact that the main difference among the experimental diets was related to the level of polyphenols included. Previous studies have similarly reported that dietary polyphenol supplementation does not necessarily produce significant effects on animal growth performance. For instance, Bešlo et al. (12) reported no relevant effects of dietary polyphenols on growth, and Flis et al. (51) observed no differences in growth performance in finishing pigs fed oat grains rich in polyphenols.

Polyphenols are primarily recognized for their biological functions related to antioxidant and anti-inflammatory activities (52, 53). These properties may contribute to improved intestinal health and overall physiological status, which could indirectly support animal performance. In the present study, although no statistically significant differences were observed between groups, the treated groups tended to show slightly improved performance indicators, particularly the HSD group. During the 101-day trial, live weight increased by approximately 57% in the HSD group, compared with 53% in the control group and 56% in the HSED group (data not shown in the table). However, these trends should be interpreted cautiously due to the absence of statistical significance.

The dietary effects observed in carcass traits were generally modest. The only statistically significant difference was the higher thigh yield observed in the HSED group compared with both the C and HSD groups. Although this result may indicate a slight tendency of the HSED diet to promote lean tissue deposition, the magnitude of the difference was small, and its biological and economic relevance for pork production is likely limited. Nevertheless, because lean cuts contribute substantially to carcass value, even minor increases may have some practical relevance, especially when thighs are destined for PDO production.

The overall limited dietary effects observed in this study may be related to the relatively low inclusion level of the by-products in the experimental diets. Such inclusion levels likely prevented negative impacts on growth performance, carcass traits, and the main commercial cuts. This finding is relevant because dietary modifications involving high levels of fibrous by-products may sometimes impair growth performance. For example, Zhu et al. (54) reported that the inclusion of 15% mulberry leaf powder, containing 11.44% crude fiber, reduced average daily gain and feed efficiency in finishing pigs. Conversely, Biondi et al. (55) observed no significant effects on growth performance when 15% tomato processing by-products were included in the diets of castrated pigs. These contrasting outcomes may be attributed to the different typologies of by-products used, their chemical composition, and processing methods, which can influence fiber structure, nutrient availability, and overall digestibility. Compared with mulberry leaf powder, tomato processing by-products and HS-derived ingredients may contain different fiber fractions and bioactive compounds, potentially mitigating negative effects on nutrient utilization and growth performance. In addition, our supplements did not interfere with growth performance, which could be due to the low level of supplementation used, given that the fiber content of HS (16.97%) is even higher than that reported for mulberry leaf powder.

Gender did not significantly influence slaughter weight or average daily gain. Nevertheless, sex-related differences were observed in the proportion of commercial cuts, indicating that physiological differences between gilts and barrows influence carcass composition. The higher yield of lean cuts observed in gilts, particularly in the loin and neck, is consistent with previous studies reporting that gilts tend to deposit less fat and produce leaner carcasses than barrows (56–58). Conversely, the higher proportion of adipose cuts observed in barrows, especially in backfat, jowl, and perirenal fat, reflects the well-known effects of castration on fat deposition patterns (59, 60). These results confirm that gender remains an important factor influencing carcass characteristics.

Meat quality parameters showed minimal variation among dietary treatments and between genders, indicating that the experimental diets did not substantially affect meat quality traits. The pH values recorded for all groups were within the normal range for pork (61). However, slightly higher pH values were observed in the treated groups, a result consistent with previous findings in finishing pigs fed diets supplemented with polyphenol-rich grape seed extract (62).

Similarly, color parameters and cooking losses showed only minor differences among treatments. The HSED group exhibited lower cooking losses at 24 h post mortem compared with the C and HSD groups (24.84% *vs* 28.3% and 26.33%, respectively), suggesting a possible improvement in water-holding capacity. Improved juiciness and reduced cooking or drip losses have previously been reported in pigs receiving plant-derived phenolic supplements or mulberry leaves in their diets (48, 54).

Regarding color parameters, our findings are consistent with several studies reporting no significant effects of dietary supplementation on meat color (63, 64). However, some studies have observed changes in the redness parameter (a*), possibly associated with alterations in myoglobin content induced by dietary antioxidants (48, 62). The effect of gender on meat color was limited. Barrows showed slightly darker meat (lower L*) at 24 h post mortem and greater color variation (higher ΔE) after 7 days of storage. Although these differences are unlikely to substantially influence consumer perception, they are consistent with previous reports indicating that barrow meat tends to be darker or redder than that of gilts (65–67). Dietary treatments did not produce statistically significant effects on the oxidative stability of muscle samples during refrigerated storage, as measured by malondialdehyde (MDA) levels. Nevertheless, lower MDA values were observed in the HSD group on day 7, particularly after cooking, suggesting a potential protective effect against lipid oxidation. Similar trends have been reported in studies evaluating HS and HSE as ingredients in pork burgers (35, 68).

However, the literature on the influence of animal diet on the oxidative stability of meat products remains inconsistent. Some studies have reported improvements in oxidative stability following dietary supplementation with antioxidant-rich ingredients, such as mulberry leaves (15%), plant-derived phenolic supplements (0.1%), or grape seed proanthocyanidin extract (50–200 mg/kg) (48, 54, 62). In contrast, other studies have found no significant effects when vegetable by-products such as tomato or bergamot processing residues were included in pig diets (55, 64).

The antioxidant activity of plant-based matrices depends not only on the concentration of bioactive compounds but also on their bioavailability, metabolic transformation during digestion, and subsequent deposition in animal tissues.

Even relatively high dietary inclusion levels do not always lead to measurable effects, whereas lower levels of other matrices may produce significant responses.

In the present study, the tendency toward lower MDA values in the treated groups may indicate that HS could act as a dietary antioxidant source. However, the absence of statistically significant effects may also be related to the limited bioavailability of polyphenols in monogastric animals. It is estimated that only 5%–10% of plant polyphenols are absorbed in the small intestine, while the remaining fraction transforms by gut microbiota into various metabolites (69). Some of these metabolites are structurally complex and may not be readily absorbed (70).

The fatty acid composition of the *longissimus thoracis* (LT) muscle and backfat (BF) is known to be influenced by the characteristics of dietary lipids (13). In this study, the inclusion of HS and HSE appeared to contribute to the preservation of PUFA content in the feeds, which was reflected in a higher PUFA proportion in the tissues of supplemented animals. Both LT and BF showed similar trends, although the effects were more pronounced in adipose tissue than in muscle. This observation is consistent with previous studies indicating that intramuscular fat in the LT is less responsive to dietary PUFA incorporation compared with adipose tissue (71, 72). Interestingly, similar trends were observed in the HSED group, even though HSE itself did not provide additional fatty acids. This result may suggest that the antioxidant compounds present in the extract contributed to protecting dietary lipids from oxidation, and this is also confirmed by the lower content of lipid hydroperoxides detected in the feed of this group compared to the feed of the control group.

Dietary polyphenols have also attracted considerable attention for their potential cholesterol-lowering effects, particularly on LDL cholesterol (73). In the present study, a reduction in cholesterol content in the LT muscle was observed in the treated groups, amounting to 7.56% and 12.10% in the HSD and HSED groups, respectively. A growing body of evidence has reported reductions in plasma LDL cholesterol following dietary polyphenol supplementation.

For example, Liu et al. (74) demonstrated that chestnut wood extract used as a source of hydrolyzable tannins in broiler diets significantly reduced plasma total and LDL cholesterol levels. Similarly, Abdulkarimi et al. (75) reported a significant decrease in plasma total and LDL cholesterol concentrations in broilers fed diets supplemented with thyme extract. Cholesterol-lowering effects of polyphenols have also been reported in laying hens, where the supplementation of high-polyphenol extra virgin olive oil significantly reduced both serum and egg yolk cholesterol levels (76).

Considering the well-established relationship between plasma and cellular cholesterol metabolism, largely mediated by LDL cholesterol (77), these mechanisms may partly explain the reductions observed in the present study. Furthermore, similar effects have been reported in pigs, where dietary supplementation with grape seed procyanidins (200–250 mg/kg) significantly reduced total cholesterol content in the LT muscle by modulating lipid metabolism (78).

Regarding gender effects, differences in fatty acid composition were observed only in the intramuscular lipids of the LT muscle. Gilts showed higher PUFA levels and lower MUFA levels compared with barrows, confirming previous observations reported in the literature (66, 79). No statistical effect of gender was detected on the cholesterol content of LT (data not shown).

## 5 Conclusion

The partial substitution of conventional feed ingredients with agro-industrial by-products may represent a strategy to improve the sustainability of animal production systems. In this study, the inclusion of hazelnut skin (HS) and its green phenolic extract (HSE) in finishing pig diets did not negatively affect growth performance, carcass characteristics, or the main meat quality parameters under the experimental conditions tested. Although most of the evaluated traits were not significantly influenced by the dietary treatments, some tendencies were observed, including slightly improved cooking losses and higher thigh yield in the HSED group, as well as lower cholesterol content in the supplemented groups. These results suggest that HS and HSE can be included in finishing pig diets at the tested levels without impairing productive performance or meat quality. Considering the limited number of studies available on the use of hazelnut by-products in pig nutrition, further research is needed to evaluate different inclusion levels and to better clarify their potential effects on animal performance, oxidative stability, and meat quality.

## Data Availability

The data presented in the study are deposited in the repository Zenodo https://doi.org/10.5281/zenodo.19630616.
